# Birds are better at regulating heat loss through their legs than their bills: implications for body shape evolution in response to climate

**DOI:** 10.1098/rsbl.2023.0373

**Published:** 2023-11-22

**Authors:** Alexandra McQueen, Ryan Barnaby, Matthew R. E. Symonds, Glenn J. Tattersall

**Affiliations:** ^1^ Centre for Integrative Ecology, School of Life and Environmental Sciences, Deakin University, Burwood, VIC 3125, Australia; ^2^ Department of Biological Sciences, Brock University, 1812 Sir Isaac Brock Way, St. Catharines, Ontario, Canada L2S 3A1

**Keywords:** Allen's rule, beak, heat exchange, infrared thermography, shape-shifting, thermoregulation

## Abstract

Endotherms use their appendages—such as legs, tails, ears and bills—for thermoregulation by controlling blood flow to near-surface blood vessels, conserving heat when it is cold, and dissipating heat in hot conditions. Larger appendages allow greater heat dissipation, and appendage sizes vary latitudinally according to Allen's rule. However, little is known about the relative importance of different appendages for thermoregulation. We investigate physiological control of heat loss via bird bills and legs using infrared thermography of wild birds. Our results demonstrate that birds are less able to regulate heat loss via their bills than their legs. In cold conditions, birds lower their leg surface temperature to below that of their plumage surface, retaining heat at their core. In warm conditions, birds increase their leg surface temperature to above that of their plumage surface, expelling heat. By contrast, bill surface temperature remains approximately 2°C warmer than the plumage surface, indicating consistent heat loss under almost all conditions. Poorer physiological control of heat loss via bird bills likely entails stronger selection for shorter bills in cold climates. This could explain why bird bills show stronger latitudinal size clines than bird legs, with implications for predicting shape-shifting responses to climate change.

## 1. Introduction

Allen's rule describes global patterns in body shape, where endotherms have larger appendages in warmer climates and at lower latitudes [1]. These patterns may be driven by thermal adaptation [2–4], where smaller appendages reduce the relative surface area available for heat loss in cold conditions, and larger appendages facilitate heat loss in warm conditions. However, many factors influence animal shape, including adaptation for foraging and locomotion [5–7]. Research investigating physiological control of heat loss from different appendages can be used to better understand climate effects on body shape evolution: among birds, geographical variation in bill size is more strongly influenced by temperature than leg size [8]. If Allen's rule in birds is explained by thermal adaptation, this difference could be explained by poorer physiological control of heat loss via bills compared to legs, leading to stronger selection for shorter bills in cold climates. However, few studies have formally compared the importance of different appendages, including bird bills and legs, for thermoregulation.

Bird bills [9–11] and legs [12–14] are effective thermal windows, used to maintain a stable core temperature; birds increase blood flow to the surface of their unfeathered bills and legs to dissipate heat in hot conditions, and restrict blood flow to conserve heat in cold conditions. Birds are expected to have a greater capacity to limit heat loss via their legs because counter-current blood vessels in limbs [15,16] allow core body heat to be conserved. Meanwhile, the proximity of a bird's bill to its brain, respiratory turbinates, and primary sense organs, could mean that blood flow to the bill is not as effectively restricted [9], leading to greater heat loss. However, contrary to these expectations, recent work demonstrates that toucans (*Ramphastos toco*) effectively minimize heat loss via their bills during cold conditions [17]. Behavioural research further suggests larger legs entail thermal costs in cold climates, since long-legged species spend more time with their legs tucked beneath their plumage than short-legged species [14].

We compared physiological control of heat loss via bird bills and legs using non-invasive, infrared thermography of wild birds (*N* = 14 species) sampled over a broad range of microclimate conditions (air temperatures 2–39°C). We further used biophysical modelling to estimate the relative propensity of bird bills and legs to expel heat.

## 2. Methods

We estimated physiological control of heat loss via bird bills and exposed legs (tarsi) by measuring changes in their surface temperature at different air temperatures. We assessed changes relative to plumage surface temperature, because feathers are not thermally regulated, and thus passively reflect recent microclimate conditions experienced by the bird. We further assessed changes in the maximum eye region surface temperature; maximum eye region temperature is the highest temperature around the bird's eye (periorbital region), where plumage is relatively sparce, and approximately indicates skin surface temperature on a body part with constant, high blood flow (electronic supplementary material, figure S1; [18]). If bills and legs are adapted for thermoregulation, we expected bill and leg surface temperatures to increase more steeply than plumage surface temperatures with increasing air temperature, reflecting the capacity to regulate heat loss through these exposed surfaces. We expected maximum eye region temperature to increase less steeply than the plumage surface with increasing air temperature, reflecting that birds maintain a stable core temperature in changing conditions.

### (a) Fieldwork

We collected thermographs from November 2020 to March 2023. Fieldwork locations were influenced by state COVID-19 restrictions; fieldwork was undertaken at Lysterfield Park, Churchill National Park, Werribee Western Treatment Plant and Point Cook Coastal Park, as well as parks and gardens in Ashwood, Ashburton, Burwood, Elsternwick, Elwood, Caufield North and Ferny Creek in Victoria, Australia.

We used a FLIR T1050sc thermal camera with an 83.4 mm focal length FLIR OSX Precision Optical System lens (FLIR Systems) to capture radiometric images and videos. Fourteen species were targeted based on their local presence, habitats and tolerance of human approach (see electronic supplementary material, figure S2 for species list and sample sizes). Thermographs of bird bills were taken in profile to minimize error associated with the angle of incidence [19,20]. Identifiable individuals were not repeatedly sampled (see electronic supplementary material, methods). For each bird, we recorded the air temperature (°C), wind speed (m s^−1^), and relative humidity (%) using a Kestrel 5000 weather meter, and solar radiation (W m^−2^) using a Digitech Solar Power Meter. Measurements were taken where the bird was sampled or, if this was practically impossible, under similar conditions nearby. Distance between the bird and the camera (1–10 m; estimated by eye to nearest 0.5 m), and relative humidity were recorded as they can affect temperature estimates [21]. We noted whether the bird was wet (yes/no) since the thermal image captures the water surface temperature rather than the bird. We assigned the same sample session ID to thermographs taken in quick succession (≤ 20 min) under similar conditions. Thermal videos enabled us to collect multiple frames in quick succession so that an in-focus frame could be selected for analysis; whether to use a video or still was decided on a case-by-case basis, depending on the bird's behaviour and field conditions.

### (b) Thermal data extraction

We extracted thermographic data using open-source image processing software Fiji [22] and ThermImageJ [23] which follows algorithms used by FLIR [24]. ThermImageJ estimates the surface temperature of objects in thermographs using radiometric data stored in each pixel and relevant emissivity, distance and microclimate information [23]. We assumed a thermal emissivity of 0.96, which is widely used for bird feathers [19,25–30]. Object distance was entered as the distance between the bird and the camera, atmospheric and reflected temperature were entered as the ambient air temperature, relative humidity was the value recorded in the field. We estimated mean surface temperatures from pixels comprising the bill, tarsus, foot and plumage-covered body, and the maximum temperature of the eye region. We excluded feathers held away from the body, wet areas and structures covering the bird. We used foot surface temperature as a proxy for tarsus surface temperature when the tarsus was not captured on the thermograph (electronic supplementary material, methods). We used eyeball and eye region areas as objective image quality filters: images with an eyeball area of less than 8 pixels or an eye region area of less than 4 pixels were excluded from analyses. This approach ensured that minimum-sized surfaces assessed were on average 20 times larger than the minimum spot size for the camera [19].

### (c) Heat loss probability

Dry heat transfer (W m^−2^) from the bill and tarsus were estimated as described by Tattersall *et al*. [10] using equations from Blaxter [31] available in the *Thermimage* R package [17]. These equations incorporate microclimate data and animal surface temperatures to estimate convective and radiative heat transfer (details in electronic supplementary material, methods). Due to uncertainty over whether birds were in thermal equilibrium, we simplified the heat transfer calculations to a categorical (yes/no) heat loss; if heat transfer from the appendage was negative, the surface was deemed to be losing heat (yes). We excluded thermographs of wet birds to focus on dry heat transfer. This allowed us to compare heat loss from bills and tarsi across differently shaped species.

### (d) Statistical analyses

We ran statistical analyses in R (v. 4.2.0; [32]). Species had a minimum *N* of 12; 10 species had *N* ≥ 40 (electronic supplementary material, figure S2).

We used Bayesian phylogenetic generalized linear mixed models (PGLMMs) (MCMC sampling with four chains of 100 000 iterations, warm-up of 20 000, thinning of 20, and weakly informative priors), run with the *brms* (v. 2.19.0) package [33] to estimate the relationship between the appendage surface temperature (response variable) and the surrounding air temperature (predictor variable) according to body surface type (interaction with air temperature). We controlled for distance, whether the bird was wet, solar radiation, and wind speed as fixed effects. We included sample session, file name, species and phylogeny as random terms to account for repeated sampling under similar conditions, within thermographs, within species, and among related species. We used a ‘maximum credible tree’ built with the *phangorn* package [34] and 10 000 phylogenetic trees from birdtree.org [35].

We assessed heat transfer as a categorical heat loss variable (yes/no) using a GLMM (family Bernouilli) with the fixed effects: body region, air temperature, solar radiation, wind speed and the interactions between body region and the three climate variables. We included the same random terms as described above. Predictors were scaled and centred to facilitate comparison between variables.

## 3. Results

### (a) Physiological control of heat loss

Across 14 species, plumage surface temperatures increased with air temperature (*b* = 1.08, *p* < 0.001), solar radiation (*b* = 0.00624, *p* < 0.001), tended to increase with increasing wind speed (*b* = 0.324, *p* = 0.074), and decreased when the bird was wet (*b* = −1.52, *p* = 0.003). Plumage surface temperatures were unaffected by observer distance (*b* = −0.0915, *p* = 0.274) and largely explained by the microclimate variables in our model (marginal *R*^2^ = 0.885; electronic supplementary material, table S1).

**Figure 1.**
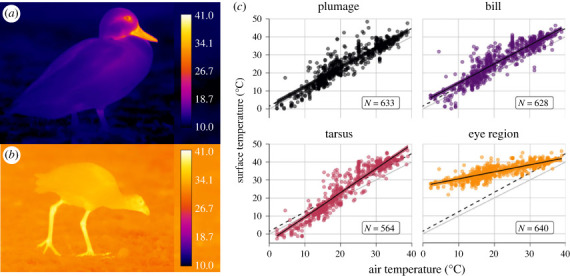
Sample thermographs showing (*a*) Pacific black duck (*Anas superciliosa*) and (*b*) purple swamphen (*Porphyrio porphyrio*); (*c*) shows surface temperatures from four body regions as a function of air temperature. Coloured lines and filled regions show PGLMM model fits ± 95% CI; points show raw temperature data; dashed line shows estimated plumage surface temperature; grey line shows equality between surface and air temperature. Sample sizes indicate the number of thermographs.

Bill surface temperature remained consistently above the plumage surface temperature (*b* = 2.34 at 0°C air temperature), increasing in parallel to the plumage surface with increasing air temperature (*b* = −0.0276, 95% CI = −0.0634, 0.00854, *p* = 0.318). Tarsus surface temperature was cooler than the plumage surface at cold air temperatures (*b* = −5.51 at 0°C air temperature) and increased more steeply than plumage surface temperature with increasing air temperature (*b* = 0.265, 95% CI = 0.229, 0.302, *p* < 0.001). Eye region surface temperature increased less steeply than plumage surface temperature with increasing air temperature (*b* = −0.689, 95% CI = −0.725, −0.654, *p* < 0.001; figure 1; electronic supplementary material, table S1).

### (b) Probability of heat loss

**Figure 2.**
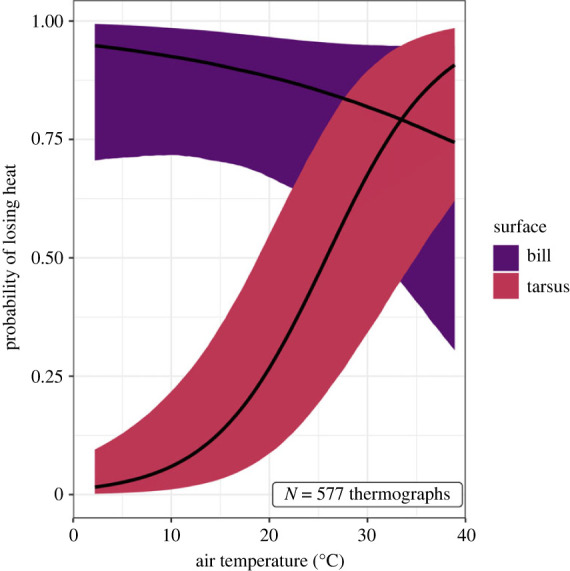
Conditional effects for the influence of air temperature on the probability of heat loss from a bird's bill and tarsus. Black lines show posterior mean, shaded areas show 95% credibility intervals. The bird's tarsus is effective at conserving heat at low temperatures, whereas the bill loses heat across all examined air temperatures.

Bird bills lost heat across all sampled air temperatures, while their tarsi had minimal heat loss at low air temperatures and a high probability of heat loss at high air temperatures (figure 2; significant interaction between appendage type and air temperature, *p* < 0.001; electronic supplementary material, table S2). The probability of heat loss from bills and tarsi decreased with increasing solar radiation and increased with increasing wind speed (electronic supplementary material, figure S4).

## 4. Discussion

Bird bills and legs are effective thermal windows, dissipating heat in hot conditions, however, birds are better able to restrict heat loss via their legs than their bills in cold conditions. At low air temperatures, leg surface temperatures were consistently below that of the plumage surface, indicating that birds actively limit warm blood flow to the limb surface, minimizing heat loss from their core. At high air temperatures, birds increase blood flow to their legs, dissipating body heat. By contrast, bird bill surface temperatures did not change significantly with air temperature but remained approximately 2°C warmer than the plumage surface, indicating constant, elevated heat loss via the bill. Maximum eye region surface temperature showed weaker changes with increasing air temperature than the plumage surface, reflective of high underlying blood flow that—unlike legs and bills—has no mechanism for reducing heat loss via vasoconstriction.

We show that blood flow to the bill is more consistently elevated compared to the legs, so that bills always lose some heat; this may be a disadvantage at low temperatures, but aids in expelling excess heat at high temperatures. Indeed recent work suggests heat dissipation via bird bills is crucial for managing heat stress, potentially mitigating the need for costly evaporative cooling [36]. Although birds can minimize heat loss via their bills by tucking their bills beneath their feathers [37,38] this may come at a cost of reduced foraging [39,40], anti-predator vigilance [41], and territorial vocalization [42], so that long bills are a liability in cold conditions. By contrast, we demonstrate effective physiological control of heat loss from bird legs, aligning with laboratory-based physiological research [9,13,43–46]; the ability of birds to minimize heat loss from their legs under cold conditions is likely related to counter-current heat exchange [15], where warm, arterial blood passes alongside cold, veinous blood flowing back to the core, exchanging heat so the leg surface becomes cold and the bird's body heat is conserved in cool environments. As environmental temperatures increase, birds increase blood flow to their legs by dilating peripheral shunt blood vessels [16,47], resulting in more warm arterial blood reaching the periphery, increasing the leg surface temperature to facilitate heat loss.

Our results suggest that longer bills are advantageous under hot conditions but likely disadvantageous under cold conditions due to consistent heat loss via the bill surface. By comparison, longer legs are advantageous in hot conditions, with relatively little thermal disadvantage in cold conditions. This difference could explain why bird bills show stronger Allen's rule effects than legs [8], especially where geographical differences in bill length are driven by adaptation to conserve heat in cold climates [7,8,48,49]. These results allow us to make further predictions for body shape evolution, including shape-shifting responses to climate warming. We expect longer legs will be more advantageous than longer bills for thermoregulation in warm conditions among temperate species that experience cold winters and hot summers, as well as migratory species adapted to contrasting climates at their breeding and non-breeding grounds. In these species, the capacity to evolve longer bills in response to climate warming may be constrained by their need to conserve heat in cold conditions. By comparison, tropical birds exposed to more consistent, mild to high temperatures are expected to show similar increases in bill and leg lengths as the climate warms.

## 5. Conclusion

Our results support a mechanistic link between global geographical patterns in appendage length and thermoregulation by demonstrating greater heat exchange per surface area via bills and legs than their plumage-covered body. Differences in physiological control of heat loss via bills and legs could explain why there are stronger Allen's rule patterns in bills compared to legs [8], since superior control of heat loss via legs can mitigate the thermal costs of long legs in cold environments.

## Data Availability

The dataset is available via the Dryad Digital Repository: https://doi.org/10.5061/dryad.7wm37pw06 [50]. Supplementary material is available online [51].

## References

[1] Allen JA. 1877 The influence of physical conditions in the genesis of species. Rad. Rev. **1**, 108-140.

[2] Alhajeri BH, Fourcade Y, Upham NS, Alhaddad H. 2020 A global test of Allen's rule in rodents. Glob. Ecol. Biogeogr. **29**, 2248-2260. (10.1111/geb.13198)

[3] Cartar RV, Morrison RIG. 2005 Metabolic correlates of leg length in breeding arctic shorebirds: the cost of getting high. J. Biogeogr. **32**, 377-382. (10.1111/j.1365-2699.2005.01237.x)

[4] McQueen A et al. 2022 Thermal adaptation best explains Bergmann's and Allen's rules across ecologically diverse shorebirds. Nat. Commun. **13**, 4727. (10.1038/s41467-022-32108-3) 35953489 PMC9372053

[5] Abourachid A, Höfling E. 2012 The legs: a key to bird evolutionary success. J. Ornithol. **153**, S193-S198. (10.1007/s10336-012-0856-9)

[6] Niemi GJ. 1985 Patterns of morphological evolution in bird genera of New World and Old-World peatlands. Ecology **66**, 1215-1228. (10.2307/1939175)

[7] Friedman NR, Miller ET, Ball JR, Kasuga H, Remes V, Economo EP. 2019 Evolution of a multifunctional trait: shared effects of foraging ecology and thermoregulation on beak morphology, with consequences for song evolution. Proc. R. Soc. B **286**, 20192474. 10.1098/rspb.2019.2474 PMC6939928 31847778

[8] Symonds MRE, Tattersall GJ. 2010 Geographical variation in bill size across bird species provides evidence for Allen's rule. Am. Nat. **176**, 188-197. (10.1086/653666) 20545560

[9] Tattersall GJ, Andrade DV, Abe AS. 2009 Heat exchange from the toucan bill reveals a controllable vascular thermal radiator. Science **325**, 468-470. (10.1126/science.1175553) 19628866

[10] Tattersall GJ, Chaves JA, Danner RM. 2018 Thermoregulatory windows in Darwin's finches. Funct. Ecol. **32**, 358-368. (10.1111/1365-2435.12990)

[11] Schraft HA, Whelan S, Elliott KH. 2019 Huffin’ and puffin: seabirds use large bills to dissipate heat from energetically demanding flight. J. Exp. Biol. **222**, jeb212563. (10.1242/jeb.212563) 31624096

[12] Maloney SK, Dawson TJ. 1994 Thermoregulation in a large bird, the emu (*Dromaius novaehollandiae*). J. Comp. Physiol. B **164**, 464-472. (10.1007/BF00714584)

[13] Steen I, Steen JB. 1965 The importance of the legs in the thermoregulation of birds. Acta Physiol. Scand. **63**, 285-291. (10.1111/j.1748-1716.1965.tb04067.x) 14324065

[14] Ryeland J, Weston MA, Symonds MRE. 2019 Leg length and temperature determine the use of unipedal roosting in birds. J. Avian Biol. **50**, e02008. (10.1111/jav.02008)

[15] Midtgård U. 1981 The rete tibiotarsale and arterio-venous association in the hind limb of birds: a comparative morphological study on counter-current heat exchange systems. Acta Zool. **62**, 67-87. (10.1111/j.1463-6395.1981.tb00617.x)

[16] Millard RW, Reite OB. 1970 Peripheral vascular response to norepinephrine at temperatures from 2 to 40 degrees C. J. Appl. Physiol. **38**, 26-30. (10.1152/jappl.1975.38.1.26) 1110238

[17] Tattersall GJ. 2021 Thermimage: Thermal Image Analysis. See http://CRAN.R-project.org/package=Thermimage

[18] Jerem P, Herborn K, McCafferty D, McKeegan D, Nager R. 2015 Thermal imaging to study stress non-invasively in unrestrained birds. J. Vis. Exp. **6**, e53184. 10.3791/53184 PMC4692699 26575985

[19] Playà-Montmany N, Tattersall GJ. 2021 Spot size, distance and emissivity errors in field applications of infrared thermography. Methods Ecol. Evol. **12**, 828-840. (10.1111/2041-210X.13563)

[20] Tabh JKR, Burness G, Wearing OH, Tattersall GJ, Mastromonaco GF. 2021 Infrared thermography as a technique to measure physiological stress in birds: body region and image angle matter. Physiol. Rep. **9**, e14865. 34057300 10.14814/phy2.14865 PMC8165734

[21] Tattersall GJ. 2016 Infrared thermography: a non-invasive window into thermal physiology. Comp. Biochem. Physiol. A Mol. Integr. Physiol. **202**, 78-98. (10.1016/j.cbpa.2016.02.022) 26945597

[22] Schindelin J et al. 2012 Fiji: an open-source platform for biological-image analysis. Nat. Methods **9**, 676-682. (10.1038/nmeth.2019) 22743772 PMC3855844

[23] Tattersall GJ. 2023 ThermimageJ—Thermal Image Functions and Macros for ImageJ. See https://github.com/gtatters/ThermImageJ.

[24] Minkina W, Dudzik S. 2009. Infrared thermography: errors and uncertainties. Chichester, UK: John Wiley & Sons, Ltd.

[25] Yahav S, Shinder D, Tanny J, Cohen S. 2005 Sensible heat loss: the broiler's paradox. World's Poultry Sci. J. **61**, 419-434. (10.1079/WPS200453) 14979577

[26] McCafferty DJ. 2013 Applications of thermal imaging in avian science. Ibis **155**, 4-15. (10.1111/ibi.12010)

[27] Greenberg R, Cadena V, Danner RM, Tattersall GJ. 2012 Heat loss may explain bill size differences between birds occupying different habitats. PLoS One **7**, e40933. (10.1371/journal.pone.0040933) 22848413 PMC3405045

[28] Tattersall GJ, Roussel D, Voituron Y, Teulier L. 2016 Novel energy-saving strategies to multiple stressors in birds: the ultradian regulation of body temperature. Proc. R. Soc. B **283**, 20161551. 10.1098/rspb.2016.1551 PMC5046907 27655770

[29] Phillips PK, Sanborn AF. 1994 An infrared, thermographic study of surface-temperature in three ratites: ostrich, emu and double wattled cassowary. J. Therm. Biol **19**, 423-430. (10.1016/0306-4565(94)90042-6)

[30] Eastick DL, Tattersall GJ, Watson SJ, Lesku JA, Robert KA. 2019 Cassowary casques act as thermal windows. Sci. Rep. **9**, 1966. (10.1038/s41598-019-38780-8) 30760849 PMC6374359

[31] Blaxter K. 1989. Energy metabolism in animals and man. Cambridge, UK: Cambridge University Press.

[32] R Core Team. 2021 R: a language and environment for statistical computing. Vienna, Austria: R Foundation for Statistical Computing. See http://www.R-project.org/.

[33] Burkner PC. 2017 brms: an R package for Bayesian multilevel models using Stan. J. Stat. Softw. **80**, 1-28. (10.18637/jss.v080.i01)

[34] Schliep K. 2011 Phangorn: phylogenetic analysis in R. Bioinformatics **27**, 592-593. (10.1093/bioinformatics/btq706) 21169378 PMC3035803

[35] Jetz W, Thomas GH, Joy JB, Hartmann K, Mooers AO. 2012 The global diversity of birds in space and time. Nature **491**, 444-448. (10.1038/nature11631) 23123857

[36] Chaves JN, Tattersall GJ, Andrade DV. 2023 Energetic costs of bill heat exchange demonstrate contributions to thermoregulation at high temperatures in toco toucans (*Ramphastos toco*). J. Exp. Biol. **226**, jeb245268. (10.1242/jeb.245268) 36752123

[37] Ryeland J, Weston MA, Symonds MRE. 2017 Bill size mediates behavioural thermoregulation in birds. Funct. Ecol. **31**, 885-893. (10.1111/1365-2435.12814)

[38] Pavlovic G, Weston MA, Symonds MRE. 2019 Morphology and geography predict the use of heat conservation behaviours across birds. Funct. Ecol. **33**, 286-296. (10.1111/1365-2435.13233)

[39] du Plessis KL, Martin RO, Hockey PAR, Cunningham SJ, Ridley AR. 2012 The costs of keeping cool in a warming world: implications of high temperatures for foraging, thermoregulation and body condition of an arid-zone bird. Glob. Change Biol. **18**, 3063-3070. (10.1111/j.1365-2486.2012.02778.x) 28741828

[40] van de Ven T, McKechnie AE, Cunningham SJ. 2019 The costs of keeping cool: behavioural trade-offs between foraging and thermoregulation are associated with significant mass losses in an arid-zone bird. Oecologia **191**, 205-215. (10.1007/s00442-019-04486-x) 31420741

[41] Carr JM, Lima SL. 2012 Heat-conserving postures hinder escape: a thermoregulation-predation trade-off in wintering birds. Behav. Ecol. **23**, 434-441. (10.1093/beheco/arr208)

[42] Luther D, Danner R. 2016 Males with larger bills sing at higher rates in a hot and dry environment. Auk **133**, 770-778. (10.1642/AUK-16-6.1)

[43] Playà-Montmany N, Gonzalez-Medina E, Cabello-Vergel J, Parejo M, Abad-Gomez JM, Sanchez-Guzman JM, Villegas A, Masero JA. 2021 The thermoregulatory role of relative bill and leg surface areas in a Mediterranean population of great tit (*Parus major*). Ecol. Evol. **11**, 15 936-15 946. (10.1002/ece3.8263) PMC8601919 34824801

[44] Lustick S. 1984 Thermoregulation in adult seabirds. In Seabird energetics (eds GC Whittow, H Rahn), pp. 183-201. Boston, MA: Springer.

[45] Martineau L, Larochelle J. 1988 The cooling power of pigeon legs. J. Exp. Biol. **136**, 193-208. (10.1242/jeb.136.1.193)

[46] Ward S, Rayner JM, Moller U, Jackson DM, Nachtigall W, Speakman JR. 1999 Heat transfer from starlings *Sturnus vulgaris* during flight. J. Exp. Biol. **202**, 1589-1602. (10.1242/jeb.202.12.1589) 10333506

[47] Midtgård U. 1980 Blood-vessels in the hindlimb of the mallard *(Anas platyrhynchos*): anatomical evidence for a sphincteric action of shunt vessels in connection with the arteriovenous heat-exchange system. Acta Zool. **61**, 39-49. (10.1111/j.1463-6395.1980.tb01289.x)

[48] Danner RM, Greenberg R. 2015 A critical season approach to Allen's rule: bill size declines with winter temperature in a cold temperate environment. J. Biogeogr. **42**, 114-120. (10.1111/jbi.12389)

[49] Fan LQ, Cai TL, Xiong Y, Song G, Lei FM. 2019 Bergmann's rule and Allen's rule in two passerine birds in China. Avian Res. **10**, 1-11. (10.1186/s40657-018-0140-7)

[50] McQueen A, Barnaby R, Symonds MRE, Tattersall GJ. 2023 Data from: Birds are better at regulating heat loss through their legs than their bills: implications for body shape evolution in response to climate. *Dryad Digital Repository*. (10.5061/dryad.7wm37pw06) PMC10663788 37990562

[51] McQueen A, Barnaby R, Symonds MRE, Tattersall GJ. 2023 Birds are better at regulating heat loss through their legs than their bills: implications for body shape evolution in response to climate. Figshare. (10.6084/m9.figshare.c.6927472) PMC10663788 37990562

